# Meta-analysis of genome-wide association studies of stable warfarin dose in patients of African ancestry^∗^

**DOI:** 10.1182/bloodadvances.2024014227

**Published:** 2024-08-22

**Authors:** Innocent G. Asiimwe, Marc Blockman, Larisa H. Cavallari, Karen Cohen, Clint Cupido, Collet Dandara, Brittney H. Davis, Barry Jacobson, Julie A. Johnson, Mohammed Lamorde, Nita A. Limdi, Jennie Morgan, Johannes P. Mouton, Sarudzai Muyambo, Doreen Nakagaayi, Arinao Ndadza, Emmy Okello, Minoli A. Perera, Elise Schapkaitz, Christine Sekaggya-Wiltshire, Jerome R. Semakula, Gayle Tatz, Catriona Waitt, Guang Yang, Eunice J. Zhang, Andrea L. Jorgensen, Munir Pirmohamed

**Affiliations:** 1 Department of Pharmacology and Therapeutics, Institute of Systems, Molecular and Integrative Biology, University of Liverpool, Liverpool, United Kingdom; 2 Division of Clinical Pharmacology, Department of Medicine, University of Cape Town, Cape Town, South Africa; 3 Department of Pharmacotherapy and Translational Research, Center for Pharmacogenomics, University of Florida College of Pharmacy, Gainesville, FL; 4 Victoria Hospital Internal Medicine Research Initiative, Victoria Hospital Wynberg, Cape Town, South Africa; 5 Division of Human Genetics, Department of Pathology, Pharmacogenomics and Drug Metabolism Research Group, Institute of Infectious Disease and Molecular Medicine, Faculty of Health Sciences, University of Cape Town, Cape Town, South Africa; 6 Department of Neurology, The University of Alabama at Birmingham, Birmingham, AL; 7 Department of Molecular Medicine and Haematology, University of the Witwatersrand, Johannesburg, South Africa; 8 Division of Pharmaceutics and Pharmacology, Center for Clinical and Translational Science, College of Medicine, The Ohio State University, Columbus, OH; 9 Infectious Diseases Institute, College of Health Sciences, Makerere University, Kampala, Uganda; 10 Metro Health Services, Western Cape Department of Health and Wellness, Cape Town, South Africa; 11 Division of Family Medicine, Department of Family, Community and Emergency Care, University of Cape Town, Cape Town, South Africa; 12 Department of Biological Sciences and Ecology, Faculty of Science, University of Zimbabwe, Harare, Zimbabwe; 13 Department of Adult Cardiology, Uganda Heart Institute, Kampala, Uganda; 14 Department of Pharmacology, Center for Pharmacogenomics, Northwestern University, Chicago, IL; 15 Department of Molecular Medicine and Hematology, Charlotte Maxeke Johannesburg Academic Hospital National Health Laboratory System Complex and University of Witwatersrand, Johannesburg, South Africa; 16 Genetics Group, Center for Applied Bioinfomatics, St. Jude Children's Research Hospital, Memphis, TN; 17 Department of Health Data Science, Institute of Population Health Sciences, University of Liverpool, Liverpool, United Kingdom

## Abstract

Warfarin dose requirements are highly variable because of clinical and genetic factors. Although genetic variants influencing warfarin dose have been identified in European and East Asian populations, more work is needed to identify African-specific genetic variants to help optimize warfarin dosing. We performed genome-wide association studies (GWASs) in 4 African cohorts from Uganda, South Africa, and Zimbabwe, totaling 989 warfarin-treated participants who reached stable dose and had international normalized ratios within therapeutic ranges. We also included 2 African American cohorts recruited by the International Warfarin Pharmacogenetics Consortium (n = 316) and the University of Alabama at Birmingham (n = 199). After the GWAS, we performed standard error-weighted meta-analyses and then conducted stepwise conditional analyses to account for known loci in chromosomes 10 and 16. The genome-wide significance threshold was set at *P* < 5 × 10^−8^. The meta-analysis, comprising 1504 participants, identified 242 significant SNPs across 3 genomic loci, with 99.6% of these located within known loci on chromosomes 10 (top SNP: rs58800757, *P* = 4.27 × 10^−13^) and 16 (top SNP: rs9925964, *P* = 9.97 × 10^−16^). Adjustment for the *VKORC1* SNP *-1639G>A* revealed an additional locus on chromosome 2 (top *SNPs rs116057875/rs115254730/rs115240773, P =* 3.64 × 10^−*8*^), implicating the *MALL* gene, that could indirectly influence warfarin response through interactions with caveolin-1. In conclusion, we reaffirmed the importance of *CYP2C9* and *VKORC1* in influencing warfarin dose requirements, and identified a new locus (*MALL*), that still requires direct evidence of biological plausibility.

## Key Points

• The *CYP2C* and *VKORC1* loci are the most important in determining warfarin dose requirements in individuals of African ancestry.
• We identified a biologically plausible locus (*MALL*) that could indirectly influence warfarin response through interactions with caveolin-1.

## Visual Abstract

**Figure:**
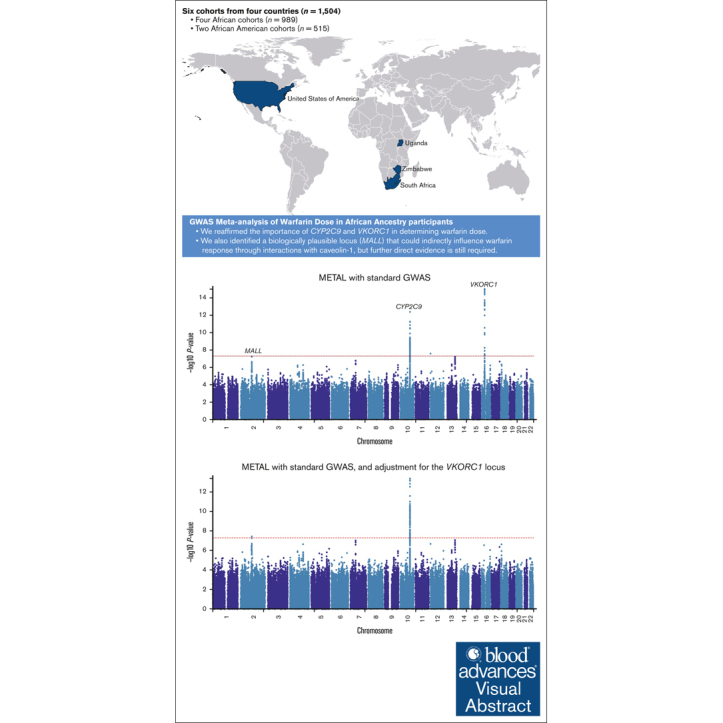

## Introduction

Warfarin remains the primary choice for oral anticoagulation therapy in resource-limited settings such as those in Africa, primarily because of its affordability.^1^ However, the continent faces challenges in achieving optimal anticoagulation,^2^ mainly because of difficulties in optimizing warfarin dosing. Its narrow therapeutic window, coupled with the significant risk of thrombotic or hemorrhagic events when doses are inadequate or excessive, respectively, contributes to warfarin being a leading cause of hospitalization because of preventable adverse drug reactions in South Africa.^3^ Moreover, there exists considerable variability in dose requirements among patients, influenced by clinical and genetic factors. Despite the development of numerous dosing algorithms incorporating these factors, Africa significantly lags behind other regions globally, with <1% of these algorithms originating from this region.^4^

Whites and Asians have been extensively studied regarding genetic variants influencing warfarin dose requirements, particularly focusing on single-nucleotide polymorphisms (SNPs) within the genes *CYP2C9* (cytochrome P450, family 2, subfamily C, polypeptide 9) and *VKORC1* (vitamin K epoxide reductase complex, subunit 1).^4^ The most studied variants, including *CYP2C9∗2* (rs1799853), *CYP2C9∗3* (rs1057910), and *VKORC1 -1639G>A* (rs9923231), have lower allele frequencies in African populations, resulting in a lesser impact on warfarin dose requirements.^5^ Therefore, more work is needed to identify additional variants that may play a more significant role in these populations. One approach to systematically explore the entire genome for such variants is through genome-wide association studies (GWASs). GWASs have been successfully conducted in a range of populations, including African Americans.^6^, ^7^, ^8^, ^9^, ^10^, ^11^ For instance, Perera et al conducted a GWAS that identified a novel variant, rs12777823, within the *CYP2C* gene region in African Americans.^6^ This variant, independent of *CYP2C9∗2* and *CYP2C9∗3*, was found to decrease the warfarin dose by 6.9 mg/wk and 9.3 mg/wk in African Americans who were heterozygous and homozygous for the A allele, respectively.

To identify rare variants or variants with small effect sizes, GWASs typically require sample sizes in the thousands.^12^ However, achieving such large sample sizes in Africa, where pharmacogenomic-related clinical research is still in its early stages, is challenging. Establishing the necessary clinical research infrastructure and ensuring accurate data capture in resource-limited settings demand significant resources.^13^ In the United States, obstacles including patient distrust of researchers and clinicians, limited knowledge about genomic research, inadequate culturally appropriate educational materials, language barriers, and economic disadvantages have hindered the recruitment and retention of racial and ethnic minorities in clinical trials.^14^ These challenges are likely to be present in Africa as well. To help overcome sample size–related issues, we conducted several GWASs for the phenotype of stable warfarin dose in patients of African ancestry (Africans and African Americans) and pooled the results using meta-analysis.

## Methods

Reporting of the study follows the STrengthening the Reporting Of Pharmacogenetic Studies guideline (supplemental Table 1).^15^

### Study design, setting, and participants

We used 6 cohorts including 4 African and 2 African American cohorts. The African cohorts were obtained through the Warfarin Anticoagulation in Patients in sub-Saharan Africa (War-PATH http://warpath.info/) observational study.^16^, ^17^, ^18^ The War-PATH study enrolled participants receiving warfarin treatment, who were on stable dose, defined as consistent prescribed doses for 2 consecutive clinic visits in the year before recruitment. Additionally, participants had to maintain an international normalized ratio (INR) within the therapeutic range (2.0-3.0 for venous thromboembolism/atrial fibrillation, and 2.5-3.5 for valvular heart disease) at both visits. The first cohort comprised 548 Black Africans recruited from 12 Ugandan and South African outpatient clinics/hospital departments between 2018 and 2020.^16^, ^17^, ^18^ The second cohort included an additional 214 Black African participants recruited from Charlotte Maxeke Johannesburg Academic Hospital, South Africa (during 2019), INR clinics at Groote Schuur Hospital and Gugulethu Community Health Centre in Western Cape, South Africa (between 2016 and 2017), and Parirenyatwa Group of Hospitals in Harare, Zimbabwe (between 2016 and 2017). Details of those recruited in South Africa and Zimbabwe between 2016 and 2017 have been previously reported.^19^^,^^20^ The third cohort consisted of 133 participants of mixed ancestry recruited from the same South African sites between 2018 and 2020. The fourth cohort comprised 94 individuals of mixed ancestry and Black Africans recruited from Uganda and South Africa between 2021 and 2022 as part of the War-PATH “bundle of care” study.^21^ The aforementioned cohorts are referred to as War-PATH cohorts 1 through 4, respectively, throughout the manuscript.

African American cohorts included the 2 cohorts analyzed by Perera et al.^6^ African Americans were recruited via specific sites by members of the International Warfarin Pharmacogenetics Consortium (IWPC, n = 316), and those recruited by the University of Alabama at Birmingham (UAB, n = 199) were recruited as part of the Pharmacogenetic Optimization of Anticoagulation Therapy study.^22^^,^^23^ Study participants in both cohorts were of self-reported African ancestry and maintained stable warfarin dosages (with various definitions, but most requiring stability for at least 3 consecutive clinic visits).^6^^,^^22^^,^^23^ The IWPC cohort was provided by IWPC researchers whereas the UAB cohort was downloaded from the Database of Genotype and Phenotype (study accession: phs000708.v1.p1). All studies adhered to relevant ethical requirements, including obtaining institutional review board approvals and individual patient informed consent.^6^^,^^16^, ^17^, ^18^, ^19^, ^20^^,^^22^^,^^23^

### Variables

We considered all SNPs (both genotyped and imputed, details provided under “Data sources/measurement”) as exposures, with the primary outcome being the stable warfarin dose, as defined in “Study design, setting, and participants.” Additionally, we accounted for 5 nongenetic predictors of stable warfarin dose: age, sex, weight, target INR range, and simvastatin/amiodarone status. These predictors were chosen based on expert guidance, literature review, and their availability across all analyzed cohorts. Although we previously included country of recruitment (a proxy of underlying population structure) as a clinical predictor of stable warfarin dose,^16^ it was omitted in this analysis. This decision was made because underlying population structure is better captured by principal components of genetic ancestry, which were available in the GWAS data sets. It is common practice in studies involving mixed ancestry participants to include the first 10 principal components to adjust for population stratification.^24^ Because some cohorts were admixed, and for consistency in analysis, we included 10 principal components of genetic ancestry as additional predictor variables in all cohorts. For analysis on the deconvolved ancestral segments (details provided in “Statistical methods” below), the proportion of specific ancestry was included as a covariate instead of the 10 principal components.

### Data sources/measurement

Details on the measurement of clinical data and DNA extraction and genotyping procedures are provided in previous reports for the included cohorts.^6^^,^^16^, ^17^, ^18^, ^19^, ^20^^,^^22^^,^^23^ Genotyping quality control (QC) and imputation processes were also as previously reported,^18^ except in this study we did not exclude participants (eg, mixed ancestry participants) who did not cluster with the 1000 genomes African populations.^25^ Briefly, participants were excluded if they had discordant sex information between clinically reported sex and sex inferred from genetic information, a genotype call rate of <95%, were related to other included participants (based on an identity-by-descent coefficient cutoff of 0.1875 in a pruned subset of uncorrelated SNPs) and had a higher amount of missingness than the related participants, and/or displayed extreme heterozygosity (identified from a plot of mean heterozygosity vs proportion of missing genotypes). SNPs were excluded if they had a minor allele frequency (MAF) of <0.01, a Hardy-Weinberg equilibrium *P* value <.000001, and/or a genotype success rate of <95%. QC analysis was performed using PLINK version 1.9,^26^ as previously documented.^18^ After QC, genotype imputation was conducted on the Michigan imputation server (https://imputationserver.sph.umich.edu/index.html#!), using the 1000 Genomes Project phase 3 reference panel (version 5, GRCh37/hg19; “AFR” population used for all cohorts, except War-PATH cohort 3 that used “Other/Mixed”),^25^ with prephasing and imputation carried out using SHAPEIT version 2^27^ and IMPUTE2^28^ software, respectively. Postimputation QC involved filtering out SNPs with an imputation quality (*R*^*2*^) of <0.3 or a MAF of <0.01.^18^

### Study power

Because we analyzed cohorts with known sample sizes, we computed study power using the power.calc.linear function (“Function to Calculate Power for Linear Models”) within the R package "genpwr."^29^ With a total sample size of 1504 (all cohorts) and assuming an additive genetic mode of inheritance, a standard deviation of stable warfarin dose of 2.66 mg/d (taken from Perera et al^6^), a minimal difference in stable dose requirements of 1 mg/d to be clinically important,^22^ and a genome-wide significance threshold of 5 × 10^−8^, study power was computed as 17.3%, 78.1%, 97.6%, and 99.8% for SNPs with MAFs of 5%, 10%, 15%, and 20%, respectively.

### Statistical methods

#### Outcome transformation

We logarithmically transformed stable warfarin dose to achieve a normal distribution and to obtain a proportional/multiplicative scale that is clinically relevant and easy to interpret.^16^^,^^30^^,^^31^

#### Handling quantitative predictors

Quantitative predictor variables were neither transformed nor categorized.

#### Missing data

We used single imputation using the Michigan imputation server^32^ (missing genotype data) and the multivariate imputation by chained equations R package^33^ (missing weight in War-PATH cohorts 1, 2, and 3), as previously described.^18^

#### GWAS

For all cohorts and following QC procedures, multivariable linear regression (using SNPTEST version 2)^34^ was undertaken assuming an additive mode of inheritance and adjustment for 5 nongenetic covariates (“Variables” subsection) and 10 principal components of genetic ancestry. For the mixed ancestry cohorts and starting with imputed quality controlled genotypes, we also conducted analysis on the deconvolved ancestral segments to obtain ancestry-specific estimates (the proportion of the specific ancestry per chromosome was adjusted for instead of the principal components). These deconvolved ancestral segments were obtained using the Tractor pipeline^24^ (available at https://github.com/eatkinson/Tractor), which includes a precursor step (“local ancestry deconvolution” using RFmix [version 2.03-r0], https://github.com/slowkoni/rfmix/blob/master/MANUAL.md)^35^ that also outputs global ancestry estimates and a step (“extracting tracts and ancestral dosages”) that produces variant call format files containing ancestry-specific genotypes. We used the same parameters reported in the Tractor manuscript^24^ except that, based on a previous report,^36^ we used 4 (African, East Asian, European, and South Asian) 1000 genome^25^ reference ancestries (instead of 2) for the sub-Saharan mixed ancestry cohorts. To inspect technical artifacts, local ancestry calls were visualized using karyogram plots produced using publicly available code (https://github.com/armartin/ancestry_pipeline).^37^

#### Meta-analysis and stepwise conditional analysis

To pool the different GWASs, we undertook standard error-weighted meta-analyses with genomic control correction using METAL (version 2011-03-25).^38^ Because conditioning on well recognized loci may help identify novel SNPs,^6^ we also undertook stepwise conditional analyses on the meta-analyzed cohorts in which we first adjusted for *VKORC1 -1639G>A* (rs9923231, number of *A* alleles), followed by the *CYP2C* cluster SNP rs12777823 (number of *A* alleles) and a composite *CYP2C9* genotype (number of the following alleles*: CYP2C9∗2*, *∗3*, *∗5*, *∗6*, *∗8*, *∗9*, and *∗11*). METAL is capable of pooling cohorts of different ethnicities (https://genome.sph.umich.edu/wiki/METAL_Documentation). Given that newer methods such as meta-regression of multi ancestry genetic association (MR-MEGA, version 0.2),^39^ which uses a principal component approach, may better account for population stratification, we also conducted a secondary meta-analysis using MR-MEGA (using 3 principal components [the maximum permitted for 6 cohorts], genomic control correction on input files, and a second genomic control correction on output files). To ensure the generalizability of results, only SNPs present in all 6 cohorts were included in the meta-analysis.

#### Downstream analysis

Genes associated with SNPs passing the nominal significance threshold were obtained from the Single Nucleotide Polymorphism Database (https://www.ncbi.nlm.nih.gov/snp/).^40^^,^^41^ Other downstream analysis (including heuristic fine-mapping to identify lead SNPs within each genomic region and expression quantitative trait loci [eQTL] analysis) were performed using the Functional Mapping and Annotation of Genome-Wide Association Studies (FUMA GWAS) platform (https://fuma.ctglab.nl/, version 1.5.2) that merges several databases and tools to facilitate genetic analysis.^42^ Key input parameters were a maximum *P* value of lead SNPs of 5 × 10^−8^, a maximum *P* value cutoff of 1 × 10^−5^, r^2^ threshold of 0.8 (used to define both independent significant and lead SNPs), 1000 genomes African populations as the reference panel, a MAF of 0.01, a maximum distance between linkage disequilibrium (LD) blocks of 250 kilobases (kb), and eQTL mapping using the Genotype-Tissue Expression (version 8) database. The statistical genome-wide significance threshold was set at *P* < 5 × 10^−8^. All statistical analysis codes are available in a published manuscript^18^ or in public repositories (eg, https://github.com/eatkinson/Tractor, https://github.com/slowkoni/rfmix/blob/master/MANUAL.md, https://github.com/armartin/ancestry_pipeline, https://genomics.ut.ee/en/tools).

## Results

### Participants

A total of 1504 participants were included in the analysis, with their demographic and clinical characteristics, stratified by cohort, outlined in Table 1. The mean age of the participants was 52.1 ± 16.4 years, with 65.4% being female. The mean proportions of African ancestry were as follows: 97.1% ± 3.4% in War-PATH cohort 1, 98.6% ± 2.7% in War-PATH cohort 2, 35.1% ± 19.2% in War-PATH cohort 3, 83.1% ± 3.0% in War-PATH cohort 4, 82.6% ± 9.2% in the IWPC cohort, and 83.7% ± 7.8% in the UAB cohort. Principal component analysis plots for all 6 cohorts are displayed in supplemental Figure 1, whereas supplemental Figures 2 and 3, show karyograms from selected War-PATH cohort 3 (the most diverse cohort) and reference samples, respectively.

**Table 1.** **Clinical/demographic characteristics of the participants included in the analysis**

	War-PATH cohort 1 (N = 548)	War-PATH cohort 2 (N = 214)	War-PATH cohort 3 (N = 133)	War-PATH cohort 4 (N = 94)	IWPC cohort (N = 316)	UAB cohort (N = 199)	Overall (N = 1504)
**Age (y)**							
Mean (SD)	46.4 (15.7)	49.0 (16.0)	60.1 (14.9)	49.3 (17.0)	57.0 (14.7)	58.9 (15.3)	52.1 (16.4)
Median [Q1, Q2]	45.6 [34.8, 57.0]	48.0 [37.0, 60.8]	62.0 [53.0, 71.0]	49.0 [36.0, 63.0]	58.0 [48.0, 67.0]	60.0 [48.0, 72.0]	52.0 [40.0, 64.0]
**Weight (kg)**∗							
Mean (SD)	75.1 (20.0)	73.3 (18.0)	77.1 (18.2)	74.8 (18.6)	93.9 (26.6)	67.3 (4.08)	77.9 (21.6)
Median [Q1, Q2]	71.4 [60.0, 86.0]	70.0 [60.0, 82.0]	74.0 [62.8, 92.0]	72.0 [61.0, 83.8]	91.8 [75.0, 107.0]	67.0 [64.0, 71.0]	72.0 [63.0, 90.0]
**Sex**							
Female	390 (71.2%)	160 (74.8%)	87 (65.4%)	39 (41.5%)	199 (63.0%)	109 (54.8%)	984 (65.4%)
Male	158 (28.8%)	54 (25.2%)	46 (34.6%)	55 (58.5%)	117 (37.0%)	90 (45.2%)	520 (34.6%)
**Ancestry proportions,**†**mean (SD)**							
African	97.1% (3.4%)	98.6% (2.7%)	35.1% (19.2%)	83.1% (3.0%)	82.6% (9.2%)	83.7% (7.8%)	86.1% (19.1%)
East Asian	0.2% (0.2%)	0.1% (0.1%)	13.2% (8.4%)	2.6% (0.8%)	-	-	1.4% (4.5%)
European	2.2% (2.7%)	1.1% (2%)	30.2% (14.2%)	8.8% (1.9%)	17.4% (9.2%)	16.3% (7.8%)	10.0% (11.5%)
South Asian	0.5% (0.8%)	0.3% (0.9%)	21.5% (10.9%)	5.4% (1.8%)	-	-	2.5% (6.9%)
**Target INR range**‡							
2.0-3.0	357 (65.1%)	163 (76.2%)	82 (61.7%)	87 (92.6%)	285 (90.2%)	199 (100%)	1173 (78.0%)
2.5-3.5	191 (34.9%)	51 (23.8%)	51 (38.3%)	7 (7.4%)	31 (9.8%)	0 (0%)	331 (22.0%)
**On simvastatin/amiodarone**							
No	497 (90.7%)	208 (97.2%)	86 (64.7%)	84 (89.4%)	238 (75.3%)	192 (96.5%)	1305 (86.8%)
Yes	51 (9.3%)	6 (2.8%)	47 (35.3%)	10 (10.6%)	78 (24.7%)	7 (3.5%)	199 (13.2%)
**Weekly dose (mg)**							
Mean (SD)	41.2 (18.5)	36.4 (14.7)	35.2 (15.4)	34.3 (11.9)	45.5 (18.7)	42.4 (18.6)	40.6 (17.8)
Median [Q1, Q2]	35.0 [30.0, 50.0]	35.0 [27.5, 42.5]	32.5 [25.0, 45.0]	32.5 [27.5, 37.5]	42.0 [32.5, 55.1]	40.0 [27.5, 50.0]	35.0 [30.0, 50.0]

MICE, multivariate imputation by chained equations; N, sample size; Q1, first quartile; Q2, second quartile; SD, standard deviation; War-PATH cohort 4, mixed ancestry and Black Africans recruited from Uganda and South Africa.

∗ 11 (2.0%), 1 (0.5%), and 7 (5.3%) participants were missing weight information in War-PATH cohorts 1 (Black Africans recruited from Uganda and South Africa), 2 (Black Africans from South Africa and Zimbabwe), and 3 (mixed ancestry South African participants), respectively, which was singly imputed using the MICE R package.

† Two and 4 reference populations used for the African American and sub-Saharan African cohorts, respectively.

‡ Those with heart valve disorders have a higher target range (2.5-3.5) than the rest (2.0-3.0) who mostly include those with atrial fibrillation and venous thromboembolism.

### SNPs

After imputation and QC, a total of 17 268 054 (War-PATH cohort 1), 16 613 526 (War-PATH cohort 2), 14 860 288 (War-PATH cohort 3), 16 854 326 (War-PATH cohort 4), 12 605 058 (IWPC cohort), and 16 987 173 (UAB cohort) SNPs were included in the GWAS analyses (supplemental Tables 2-11).

### Meta-analyses

**Figure 1.**
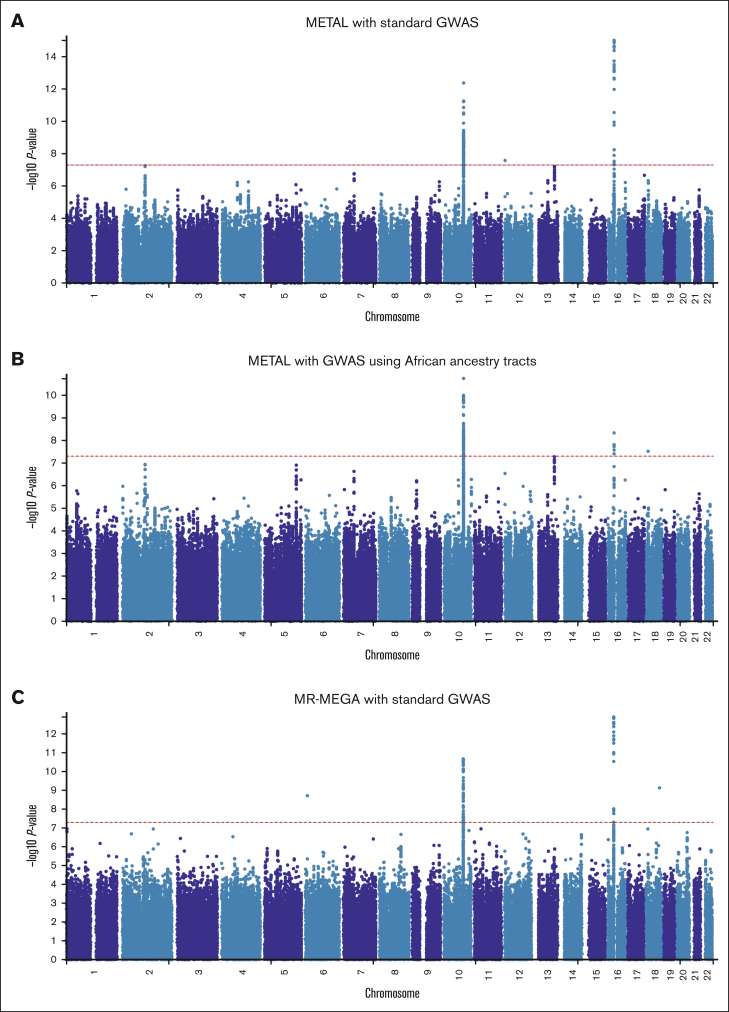
**Manhattan plots of the association between SNPs and stable warfarin dose after pooling 6 African ancestry cohorts in a meta-analysis (n = 1504 participants).** Individual GWAS analyses were undertaken using logarithm transformed stable warfarin dose, adjusted for age, sex, weight, target INR range, simvastatin/amiodarone status, and either the first 10 principal components of genetic ancestry or the proportion of the specific ancestry per chromosome by frequentist association testing assuming an additive model of inheritance before being pooled using METAL or MR-MEGA. (A) METAL meta-analysis with standard GWAS (242 GWAS-significant SNPs [3 genomic loci] and a genomic inflation factor of 1.023). (B) METAL meta-analysis with GWAS using African ancestry tracts (181 GWAS-significant SNPs [3 genomic loci] and a genomic inflation factor of 1.027). (C) MR-MEGA meta-analysis with standard GWAS (119 GWAS-significant SNPs [4 genomic loci] and a genomic inflation factor of 1.000). The red horizontal lines represent the genome-wide (5 × 10^−8^) significance threshold.

**Figure 2.**
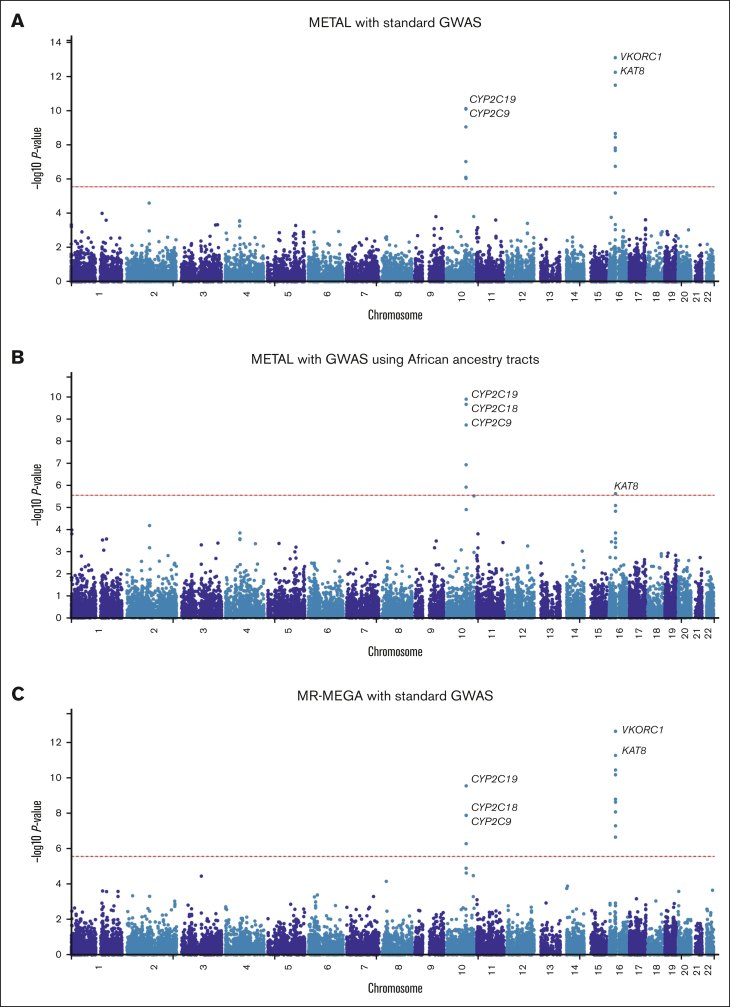
**Manhattan plots of the association between SNPs and stable warfarin dose after pooling 6 African ancestry cohorts in a meta-analysis, gene-based analysis (n = 1504 participants).** Individual GWAS analyses were undertaken using logarithm transformed stable warfarin dose, adjusted for age, sex, weight, target INR range, simvastatin/amiodarone status, and either the first 10 principal components of genetic ancestry or the proportion of the specific ancestry per chromosome by frequentist association testing assuming an additive model of inheritance before being pooled using METAL or MR-MEGA. The red horizontal lines represent the Bonferroni corrected significance thresholds (0.05 divided by the number of protein coding genes) and included 2.7 × 10^−6^ (18 244 genes), 2.7 × 10^−6^ (18 231 genes), and 2.7 × 10^−6^ (18 244 genes) for (A) METAL meta-analysis with standard GWAS, (B) METAL meta-analysis with GWAS using African ancestry tracts, and (C) MR-MEGA meta-analysis with standard GWAS, respectively. Some top genes are annotated. *CYP2C9/18*, cytochrome P450, family 2, subfamily C, polypeptide 9/18; *KAT8*, lysine acetyltransferase 8; *VKORC1*, vitamin K epoxide reductase complex, subunit 1.

In the first meta-analysis (Figure 1A; supplemental Table 12) that pooled the results obtained from the standard GWAS (n = 1504) using METAL, 242 SNPs across 3 loci passed the genome-wide significance threshold and included loci on chromosome 10 (215 SNPs, lead SNP rs58800757, *P* = 4.27 × 10^−13^, average MAF = 0.33), chromosome 12 (1 SNP, rs3794303, *P* = 2.66 × 10^−8^, MAF = 0.12), and chromosome 16 (26 SNPs, lead SNP rs9925964, *P* = 9.97 × 10^−16^, MAF = 0.14). When only the African tracts from the mixed ancestry cohorts were included in the meta-analysis (Figure 1B; supplemental Table 13), the number of genome-wide significant SNPs slightly decreased to 181 with the number of loci remaining similar: chromosome 10 (173 SNPs, lead SNP rs58800757, *P* = 1.84 × 10^−11^, MAF = 0.37), chromosome 16 (7 SNPs, lead SNP rs9925964, *P* = 4.69 × 10^−9^, MAF = 0.08), and chromosome 18 (1 SNP, rs192807508, *P* = 3.02 × 10^−8^, MAF = 0.04). In the secondary meta-analysis that used MR-MEGA (Figure 1C; supplemental Table 14), 119 SNPs across 4 loci passed the genome-wide significance threshold and included: chromosome 6 (1 SNP, rs55952617, *P* = 1.90 × 10^−9^, MAF = 0.12), chromosome 10 (96 SNPs, lead SNP rs34582766, *P* = 2.10 × 10^−11^, MAF = 0.27), chromosome 16 (21 SNPs, lead SNP rs9934438, *P* = 1.25 × 10^−13^, MAF = 0.09), and chromosome 18 (1 SNP, rs17080365, *P* = 7.29 × 10^−10^, MAF = 0.04). Corresponding gene-based meta-analyses (Figure 2) revealed only 2 significant loci on chromosomes 10 and 16, which include the well-established warfarin genes *CYP2C9* (involved in warfarin metabolism) and *VKORC1* (the molecular target of warfarin), respectively. Another observation in Figures 1 and 2 is that for a meta-analysis using the standard GWAS, the most statistically significant locus is in chromosome 16, but this changes to chromosome 10 when only African ancestry tracts are pooled. The quantile-quantile plots of both the SNP- and gene-based meta-analyses are shown in supplemental Figure 4.

**Figure 3.**
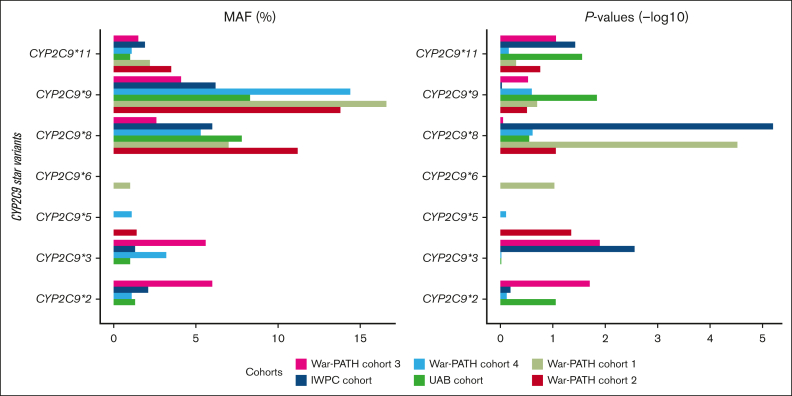
**MAFs and *P* values for the *CYP2C9* star variants across the 6 cohorts.** The left and right panels respectively show the MAFs and negative log-transformed *P* values for the 7 *CYP2C9* star variants. SNPs with MAFs of <1% were not analyzed in the respective cohorts. The cohorts are arranged by the proportion of African ancestry: War-PATH cohort 3 comprising 133 mixed ancestry South African participants (35.1% African ancestry); IWPC cohort comprising 316 African Americans recruited from USA (82.6% African ancestry); War-PATH cohort 4 comprising 94 mixed ancestry and Black Africans recruited from Uganda and South Africa (83.1% African ancestry); UAB cohort comprising 199 African Americans recruited from USA (83.7% African ancestry); War-PATH cohort 1 comprising 548 Black Africans from Uganda and South Africa (97.1% African ancestry); and, War-PATH cohort 2 comprising 214 Black Africans recruited from South Africa and Zimbabwe (98.6% African ancestry). *CYP2C9*, cytochrome P450, family 2, subfamily C, polypeptide 9.

Supplemental Table 15 shows MAFs and *P* values for the well-established *CYP2C* gene cluster (including *CYP2C9*), *CYP4F2*, and *VKORC1* warfarin-related SNPs in the individual cohorts, whereas Table 2 shows the pooled MAFs, effect-sizes, and *P* values for the SNPs present in all the 6 cohorts. The SNPs that passed the genome-wide significance threshold include 1 in *CYP2C9* (*CYP2C9∗8*, rs7900194, lowest *P* = 1.63 × 10^−9^), 2 in *VKORC1* (*VKORC1 1173C>T*, rs9934438, lowest *P* = 1.21 × 10^−15^; *VKORC1 -1639G>A*, rs9923231, lowest *P* = 1.24 × 10^−15^), and 1 in the *CYP2C* cluster (rs12777823, lowest *P* = 7.94 × 10^−11^). Figure 3 visually presents the MAFs and *P* values for *CYP2C9*, highlighting the relative importance of the different star alleles across the 6 populations. For example, War-PATH cohort 3 with the lowest proportion of African ancestry (35%) and the highest proportion of European ancestry (30%) had the highest *CYP2C9∗*2 MAF (6%) and the most significant *CYP2C9∗*2 *P* value (*P* = .02).

**Table 2.** **Effect sizes and *P* values for the well-established warfarin-related SNPs**

rsID (reference/alternative alleles)	Common name	CHR	Position∗	All cohorts (METAL)						All cohorts (African tracts, METAL)						All cohorts (MR-MEGA)†		
				Common MAF (%)	β‡ (SE)	*P*	Direction§	Het *I*^*2*^	Change in warfarin dose‖ (%)	Common MAF (%)	β‡ (SE)	*P*	Direction§	Het *I*^*2*^	Change in warfarin dose‖ (%)	Common MAF (%)	*P*	Direction§
rs7900194 (G/A)	*CYP2C9∗8*	10	96702066	0.075	−0.421 (0.070)	***1.63E−09***	−−+−−−	36.8	34.4	0.081	−0.365 (0.064)	***9.19E−09***	−−−−−−	0.0	30.6	0.070	8.62E−08	−−+−−−
rs2256871 (A/G)	*CYP2C9∗9*	10	96708974	0.134	−0.141 (0.054)	9.29E−03	−−−−−−	0.0	13.1	0.137	−0.119 (0.051)	1.93E−02	−−−−+−	0.0	11.2	0.117	1.18E−01	−−−−−−
rs28371685 (C/T)	*CYP2C9∗11*	10	96740981	0.023	−0.393 (0.126)	1.77E−03	−−−−−−	0.0	32.5	0.028	−0.286 (0.110)	9.64E−03	−−−−−−	0.0	24.9	0.020	1.16E−02	−−−−−−
rs12777823 (G/A)	*rs12777823 (G>A)*	10	96405502	0.274	−0.229 (0.039)	***4.86E−09***	−−++−−	75.7	20.5	0.284	−0.230 (0.037)	***7.75E−10***	−−+−−−	38.7	20.5	0.274	***7.94E−11***	−−++−−
rs7294 (G/A)	*VKORC1 3730G>A*	16	31102321	0.470	0.148 (0.036)	3.60E−05	++++++	68.8	15.9	0.473	0.089 (0.035)	9.56E−03	+++−++	0.4	9.4	0.471	1.96E−06	++++++
rs2359612 (C/T)	*VKORC1 2255C>T*	16	31103796	0.240	0.191 (0.041)	3.01E−06	+++−++	62.6	21.0	0.207	0.115 (0.042)	6.00E−03	+++−++	0.0	12.2	0.233	8.21E−07	+++−++
rs8050894 (G/C)	*VKORC1 1542G>C*	16	31104509	0.256	−0.211 (0.041)	2.63E−07	−−−−−−	60.4	19.0	0.229	−0.106 (0.041)	1.03E−02	−−−−+−	0.0	10.0	0.248	1.52E−07	−−−−−−
rs9934438 (C/T)	*VKORC1 1173C>T*	16	31104878	0.141	−0.495 (0.062)	***1.21E−15***	−−−−−−	0.0	39.0	0.052	−0.449 (0.080)	***1.56E−08***	−−−−−−	9.7	36.2	0.093	***1.25E−13***	−−−−−−
rs9923231 (G/A)	*VKORC1 -1639G>A*	16	31107689	0.141	−0.496 (0.062)	***1.24E−15***	−−−−−−	0.0	39.1	0.052	−0.450 (0.080)	***1.60E−08***	−−−−−−	9.4	36.2	0.093	***1.29E−13***	−−−−−−

Data shown for n = 1504. Only SNPs present in all 6 cohorts were pooled during meta-analysis.

Gene names are italicized whereas *P* values passing the genome-wide significance threshold of *P* < 5 × 10^−8^ are italicized and bolded.

CYP, cytochrome P450; Het *I*^*2*^, heterogeneity *I*^*2*^ statistic; NA, not applicable; rsID, reference SNP cluster ID; SE, standard error; *VKORC1*, vitamin K epoxide reductase complex, subunit 1.

∗ Human assembly GRCh37 (hg19).

† No β (SE) and Het *I*^*2*^ values reported for MR-MEGA.

‡ Coefficient of the alternative allele relative to the reference allele.

§ Each symbol represents a cohort in the order War-PATH cohort 1, War-PATH cohort 2, War-PATH cohort 3, War-PATH cohort 4, IWPC cohort, and UAB cohort (see Table 1; supplemental Table 15 for details). The “+” and “−“ directions indicate increased and decreased weekly warfarin doses, respectively, in the individual included cohorts.

‖ Computed from the log-transformed betas using the formula: absolute(exp(β) − 1) × 100. A corresponding negative (positive), β means the variant will decrease (increase) weekly warfarin dose by the computed percentage.

**Figure 4.**
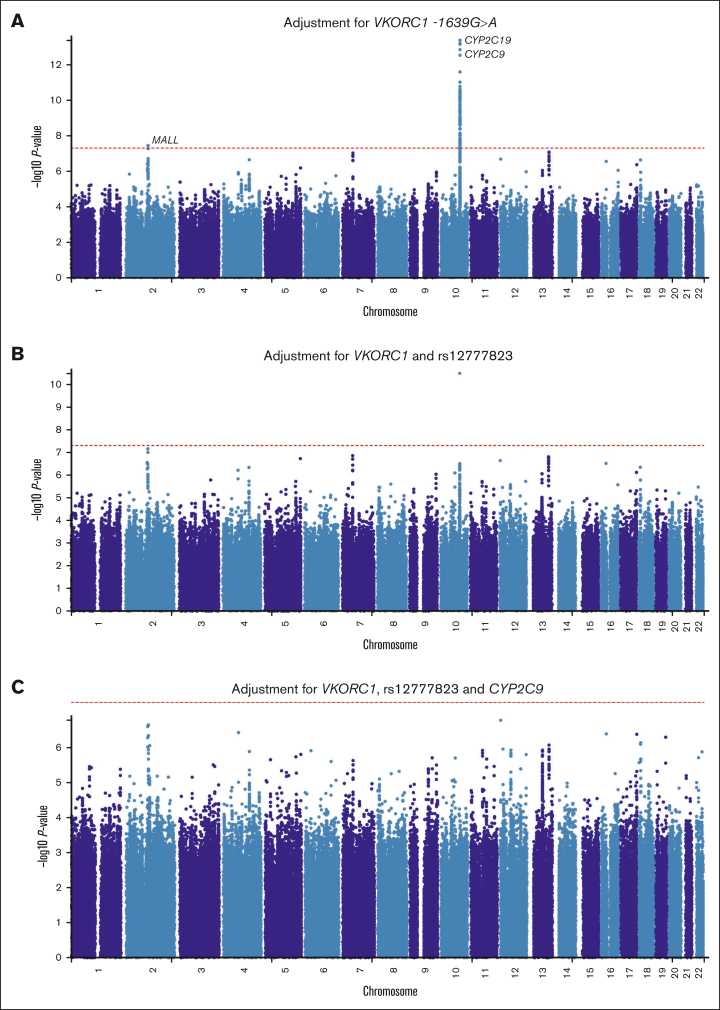
**Manhattan plots of the association between SNPs and stable warfarin dose after pooling 6 African ancestry cohorts in a meta-analysis (n = 1504) and conditioning for well-established loci.** Individual GWAS analyses were undertaken using logarithm transformed stable warfarin dose, adjusted for age, sex, weight, target INR range, simvastatin/amiodarone status, and either the first 10 principal components of genetic ancestry or the proportion of the specific ancestry per chromosome by frequentist association testing assuming an additive model of inheritance before being pooled using METAL. (A) Adjustment for *VKORC1 -1639 G>A* (genomic inflation factor = 1.023). (B) Adjustment for *VKORC1* and rs12777823 (genomic inflation factor = 1.022). (C) Adjustment for *VKORC1*, rs12777823 and *CYP2C9* (genomic inflation factor = 1.020). The red horizontal lines represent the genome-wide (5 × 10^−8^) significance thresholds. The top genes (obtained from a FUMA-GWAS [platform] gene-based Manhattan plot) per main loci are annotated. *CYP2C9/18*, cytochrome P450, family 2, subfamily C, polypeptide 9/18.

To identify potential novel loci, we adjusted our analysis for the well-established chromosome 10 and 16 loci that influence warfarin dose requirements. The results of these adjustments using METAL are shown in Figure 4, supplemental Figure 5, and supplemental Tables 16-18. Specifically, after adjusting for (1) *VKORC1 -1639G>A*, (2) *VKORC1 -1639G>A* and rs12777823, and (3) *VKORC1 -1639G>A*, rs12777823, and compound *CYP2C9* genotype (number of the following alleles*: CYP2C9∗2*, *∗3*, *∗5*, *∗6*, *∗8*, *∗9*, and *∗11*) loci, we identified 2 (on chromosomes 2 and 10), 1 (chromosome 10), and no significant loci, respectively. Of note, the adjustment for *VKORC1* alone unveiled a locus (supplemental Figure 6) on chromosome 2 (top SNPs rs116057875, rs115254730, and rs115240773 [in perfect LD], *P* = 3.64 × 10^−8^, *MALL* [mal, T-cell differentiation protein like] introns). These SNPs had an effect size (β) of 0.4053 (standard error, 0.0736), which means each mutant allele increased weekly warfarin dose by an average of 50% (using the formula: absolute(exp(β) − 1) × 100). However, none of these SNPs had significant eQTLs (supplemental Figure 6). Additionally, in a secondary meta-analysis using MR-MEGA (supplemental Table 19), this locus was not detected. Instead, 3 new loci were identified: 1 on chromosome 13 (3 SNPs in the *STK24* [serine/threonine kinase 24] gene), 1 on chromosome 14 (3 intergenic SNPs), and 1 on chromosome 17 (1 SNP in the *TSPOAP1* [TSPO associated protein 1] gene). Because of this discrepancy and because METAL does not directly adjust for population stratification, we repeated the METAL meta-analysis using only the 5 cohorts with at least 80% African ancestry (n = 1371; supplemental Table 20). The chromosome 2 locus, represented by the intergenic SNP rs114960663 (*P* = 4.96 × 10^−8^), was 1 of 3 detected loci. The *MALL* SNPs were an average of 111 kb from this SNP, placing them within the same locus (defined as a maximum distance of 250 kb between LD blocks).

## Discussion

Patients of African ancestry are often underrepresented in pharmacogenomic research, primarily because of challenges in study recruitment.^13^^,^^18^ Additionally, the considerable genetic diversity within these populations necessitates large sample sizes for well-powered studies.^13^^,^^43^ To address these issues, we conducted a meta-analysis combining data from 1504 patients treated with warfarin across 4 African and 2 African American cohorts. In this pooled analysis, we identified 242 SNPs that reached genome-wide significance (*P* < 5 × 10^−8^). Of these, 99.6% (241/242) were within known loci on chromosomes 10 (*CYP2C9*, involved in warfarin metabolism) and 16 (*VKORC1*, warfarin’s molecular target).

To identify potential new genetic loci, we adjusted our analysis for known loci, including *VKORC1 -1639G>A*, rs12777823, and compound *CYP2C9* genotype. Adjusting for *VKORC1* revealed a potential new locus on chromosome 2 within the *MALL* gene. None of the 3 intronic variants (rs116057875, rs115254730, and rs115240773) in this gene was associated with any literature in the National Library of Medicine’s National Centre for Biotechnological Information SNP database as of 14 June 2024, nor were they implicated in gene regulation during eQTL analysis. Additionally, these variants were not reported in the GWAS catalog, as queried through the FUMA GWAS platform. According to National Library of Medicine’s National Centre for Biotechnological Information’s gene database, the protein encoded by *MALL* is involved in raft-mediated trafficking in endothelial cells and localizes in glycolipid- and cholesterol-enriched membrane rafts. The integrity of endothelial cells (known to control the hemostatic pathway),^44^ is regulated by the caveolin-1 membrane protein.^45^ Given that the *MALL* encoded protein interacts with caveolin-1,^46^ an indirect influence of *MALL* on warfarin's anticoagulant action is biologically plausible. However, in a secondary meta-analysis using MR-MEGA instead of METAL, the *MALL* locus was not detected. Whereas MR-MEGA uses metaregression incorporating ancestry principal components to account for population structure in multiethnic GWASs,^39^ it can systematically detect weaker associations when risk loci confer relatively homogeneous risk across ancestries.^47^ This could explain the increase in the *P* value from *P* = 3.64 × 10^−8^ (METAL meta-analysis; heterogeneity *I*^*2*^ = 0%) to *P* = 4.24 × 10^−6^ (MR-MEGA meta-analysis). This observation was not unique to the *MALL* locus but was also observed for other SNPs such as *VKORC1* -1639G>A (heterogeneity *I*^*2*^ = 0% in a standard GWAS METAL meta-analysis, Table 2). In cases in which allelic effect heterogeneity exists between cohorts (eg, rs12777823, which has been reported to influence warfarin dose only in patients of African ancestry),^6^ MR-MEGA demonstrates the highest power.^39^^,^^47^ This is consistent with the decrease in *P* value for rs12777823 from *P* = 4.86 × 10^−9^ (METAL meta-analysis; heterogeneity *I*^*2*^ = 76%) to *P* = 7.94 × 10^−11^ (MR-MEGA meta-analysis). Given MR-MEGA’s limitations and the fact that this locus was still significant when we included only the 5 cohorts with >80% African ancestry, the influence of the biologically plausible *MALL* SNPs cannot be discounted, warranting further examination. Another issue that questions *MALL*’s significance is why it only became significant after conditioning on the *VKORC1* locus. This highlights the importance of balancing covariate inclusion: adding covariates can reduce power by increasing model complexity, especially with small sample sizes, unless the covariates explain significant variability in the outcome, thus reducing residual variance. In the case of *MALL*, the *P* value decreases from *P* = 6.14 × 10^−8^ to *P* = 3.64 × 10^−8^ when adjusting for *VKORC1*. However, stepwise additions of rs12777823 and *CYP2C9* increase the *P* values to 7.01 × 10^−8^ and 2.21 × 10^−7^ respectively, which may indicate that the variance explained by these covariates is offset by the increased model complexity, resulting in less power. Larger studies or more direct evidence of *MALL*’s influence on warfarin dose are needed to clarify its importance.

Excluding admixed individuals from GWASs may be unacceptable from both ethical (may exacerbate health inequalities) and scientific (some cardiovascular disorders are more common in admixed individuals) perspectives.^24^^,^^48^, ^49^, ^50^ We therefore included 4 admixed cohorts in our analysis. Among these, 3 cohorts (War-PATH cohort 4, IWPC cohort, and UAB cohort) had relatively high mean African ancestry proportions (∼83%), whereas the fourth cohort (War-PATH cohort 3) had a more diverse ancestry profile, including African (35%), East Asian (13%), European (30%), and South Asian (22%) ancestries, which is expected in some South African populations.^36^ To account for admixture, we used Tractor GWAS to deconvolute ancestral tracts/segments^24^; when only African tracts were pooled, the results were consistent with those of standard GWAS meta-analyses, identifying 2 main loci associated with *CYP2C9* and *VKORC1*. However, the relative importance of these loci shifted, with *CYP2C9* becoming more significant compared with *VKORC1*. This shift can be attributed to the higher influence of *VKORC1*'s key variant, *VKORC1 -1639G>A*, in European ancestries because of higher MAFs (0.39 vs 0.05 in the 1000 genomes populations).^25^ An examination of supplemental Table 15 indeed shows that local ancestry deconvolution before Tractor GWAS changed the *VKORC1 -1639G>A* MAF from 0.30 to 0.05 (War-PATH cohort 3), 0.12 to 0.08 (War-PATH cohort 4), 0.10 to 0.03 (IWPC cohort), and 0.10 to 0.06 (UAB cohort), which shifted *VKORC1*’s importance. Importantly, despite this shift, *VKORC1* remained clinically relevant in patients with exclusively African ancestry.

It is now recognized that the *CYP2C9* alleles *∗2* and *∗3*, more common in European populations (with MAFs of 0.12 and 0.07 respectively) but rarer in African populations (both <0.01),^25^ may have less impact in Africans (at population level) compared with other *CYP2C9* alleles such as *∗5*, *∗6*, *∗8*, and *∗11*.^5^^,^^51^^,^^52^ In Figure 3, we illustrate how these alleles vary in importance across the 6 cohorts. For instance, *CYP2C9∗2* and *∗3* were not assessed in the predominantly African War-PATH cohorts 1 and 2 because of their very low MAFs (<1%). Compared with other cohorts, War-PATH cohort 3 had the highest European ancestry (30%) and lowest African ancestry (35%); in this cohort, both alleles had the highest MAFs (∼0.06), resulting in relatively low *P* values (0.02 and 0.01, respectively). The IWPC cohort (17% European ancestry) showed a more significant *CYP2C9∗3 P* value (*P* = .003), likely because of its larger sample size (IWPC, 316; War-PATH cohort 3, 133). As recommended by the Association for Molecular Pathology and the College of American Pathologists, genotyping panels for all, including African, populations should, at a minimum, include the *CYP2C9* tier 1 alleles *∗2*, *∗3*, *∗5*, *∗6*, *∗8*, and *∗11*.^51^ Lastly, observed MAFs align with weighted averages of reference/ancestral populations. For instance, the 1000 genomes data indicates *CYP2C9∗2* MAFs of 0.008 for Africans, 0.001 for East Asians, 0.124 for Europeans, and 0.035 for South Asians.^25^ When weighted by the mean proportion of each ancestry in War-PATH cohort 3 (35% African, 13% East Asian, 30% European, and 22% South Asian), the weighted MAF for *CYP2C9∗2* is 0.048, aligning with the reported MAF of 0.06 in this study. Similarly, for *CYP2C9∗3*, with MAFs of 0.002 (African), 0.034 (East Asian), 0.073 (European), and 0.109 (South Asian), the weighted average is 0.051, which is comparable with the MAF of 0.056 in our study.

Our study’s main limitation lies in its limited statistical power and the lack of a replication cohort. Updated power calculations using the observed warfarin dose standard deviation of 17.8 mg/wk (Table 1), a clinically important effect size of 7 mg/wk,^22^ and a *P* value of 5 × 10^−8^ revealed that with a sample size of 1504 participants, we had 23% power to detect SNPs with MAFs of 5% (86% power for SNPs with MAFs of 10%). The 7 mg/wk effect size corresponds to 20% of a 35 mg/wk dose, meaning a 50% change in dose (as seen for the *MALL* SNPs) would correspond to an effect size of 17.5 mg/wk. This would result in 98% power to detect SNPs with MAFs as low as 1%, given a sample size of 1504 participants. However, SNPs with large effect sizes are rare, which means our study was underpowered to detect SNPs with moderate effect sizes and low MAFs, which are common in diverse populations. Future studies aiming to identify such SNPs should prioritize larger sample sizes. To try to address this limitation, we included mixed ancestry cohorts and used the Tractor pipeline, which can enhance power by detecting ancestry-specific signals.^24^ However, like MR-MEGA,^39^^,^^47^ Tractor's power is optimized when allele effect sizes vary by ethnicity.^24^^,^^53^ As a result, some Tractor analyses yielded less significant results, especially when the effect sizes were consistent across ancestries. Additionally, we did not apply corrections for multiple testing or use a more stringent significance threshold in our Tractor analyses. For instance, the appropriate *P* value threshold for Tractor associations is estimated at 1 × 10^−8^ for 2-way admixture,^24^ which becomes even more conservative with 4-way admixture, potentially leading to some false positive findings. Furthermore, the imputation quality may have been influenced by the imputation panels used, which included populations from the 1000 Genomes Project representing African or mixed/other groups. However, it is worth noting that 2 of the *MALL* SNPs were directly genotyped in the War-PATH cohorts and had imputation quality *R*^*2*^ values of 94% and 97% in the IWPC and UAB cohorts, respectively. Despite these limitations, our study represents, to our knowledge, 1 of the first GWAS investigating warfarin dose in sub-Saharan Africa, and the inclusion of diverse populations from both sub-Saharan Africa and North America increases the generalizability of our findings.

In conclusion, we report a meta-analysis of 1504 of African ancestry patients treated with warfarin across 4 African and 2 African American cohorts. This study reinforced the significant roles of *CYP2C9* (involved in warfarin metabolism) and *VKORC1* (the molecular target of warfarin) in influencing warfarin dose requirements. We also identified a new locus (*MALL*), that still requires direct evidence of biological plausibility. This study emphasizes the need for larger warfarin-related pharmacogenetic studies of patients of African ancestry to identify African-specific genetic variants, and ultimately improve the quality of anticoagulation in this understudied patient population.

Conflict-of-interest disclosure: M.P. currently receives partnership funding for the following: MRC Clinical Pharmacology Training Scheme (cofunded by the 10.13039/501100000265Medical Research Council and Roche, Union Chimique Belge [UCB] Pharma, Eli Lilly, and Novartis); has developed an HLA genotyping panel with MC Diagnostics but does not benefit financially from this; and is part of the Innovative Medicines Initiative Consortium Accelerating Research and Development for Advanced Therapies (www.ardat.org). C.W. is supported by a Wellcome Clinical Research Career Development Fellowship 222075/Z/20/Z. None of these of funding sources have been used for the current paper. The remaining authors declare no competing financial interests.

## Supplementary Material

Supplemental Figures and References
