# Supplementary material for: Meta-analysis of genome-wide association studies of stable warfarin dose in patients of African ancestry
Source: Blood Adv. 2024 Aug 22;8(20):5248–61. doi: 10.1182/bloodadvances.2024014227 (PMC11493193; doi:10.1182/bloodadvances.2024014227)
Supplement: Supplemental Figures and References [file BLOODA_ADV-2024-014227-mmc1.pdf]

Meta-analysis of genome-wide association studies of stable warfarin dose in patients of black-African ancestry.

Table of contents

Supplemental Figures……………………………………………………………………………………………………….2

Supplemental References…………………………………………………………………………………………………8

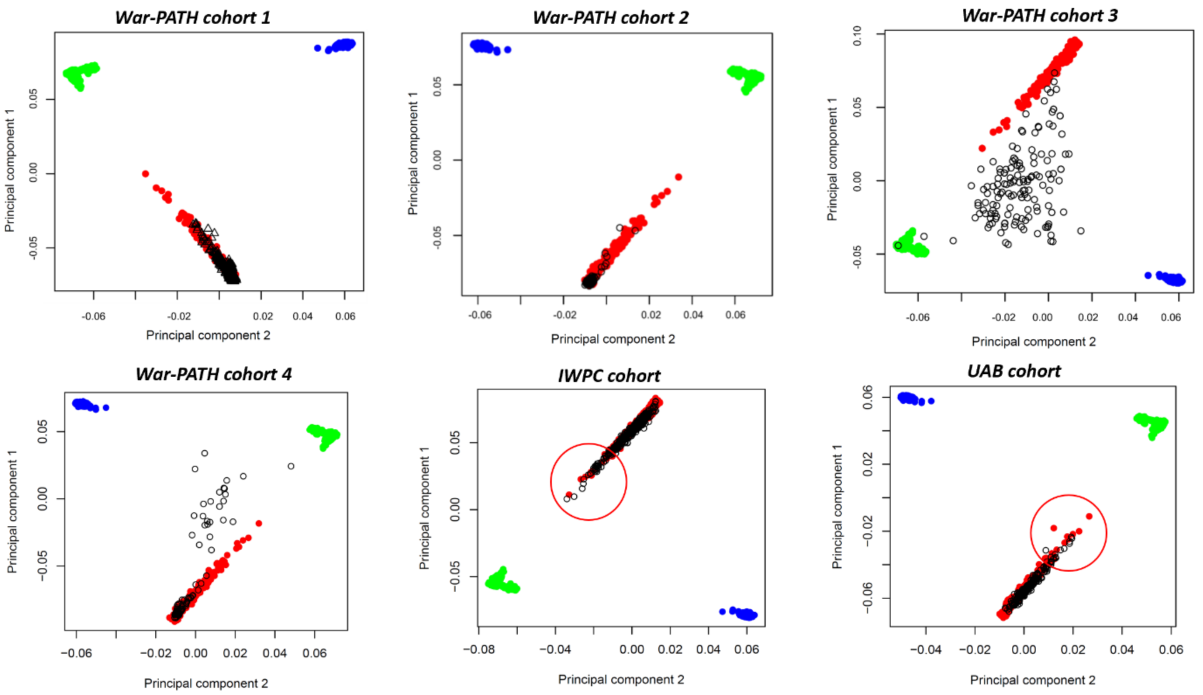

Supplemental Figure 1. Principal component analysis (PCA) plots of the analysed cohorts. All black-African cohorts (War-PATH cohorts 1 and 2) clustered with the 1000 genomes African populations,[1] as did the two African American (IWPC and UAB) cohorts. The 1000 genomes African populations include admixed individuals (red circles in the figure above; specifically, these were African Caribbeans from Barbados) and so these were excluded during ancestry deconvolution for more homogeneous reference populations. Since South Africa also has individuals with South Asian ancestry,[2] this was also included as a reference population (Figure S2) during the ancestry deconvolution for these participants. Study samples = open circles/triangles, Africans (1000 genomes) = RED, Europeans (1000 genomes) = Green, and East Asians (1000 genomes) = Blue. IWPC = International Warfarin Pharmacogenetics Consortium, UAB = University of Alabama at Birmingham, War-PATH = WARfarin anticoagulation in PATients in Sub-Saharan Africa, War-PATH cohort 1 = 548 black-Africans recruited from Uganda and South Africa, War-PATH cohort 2 = 214 black-Africans from South Africa and Zimbabwe, War-PATH cohort 3 = 133 mixed-ancestry South African participants, War-PATH cohort 4 = 94 mixed-ancestry and black-Africans recruited from Uganda and South Africa.

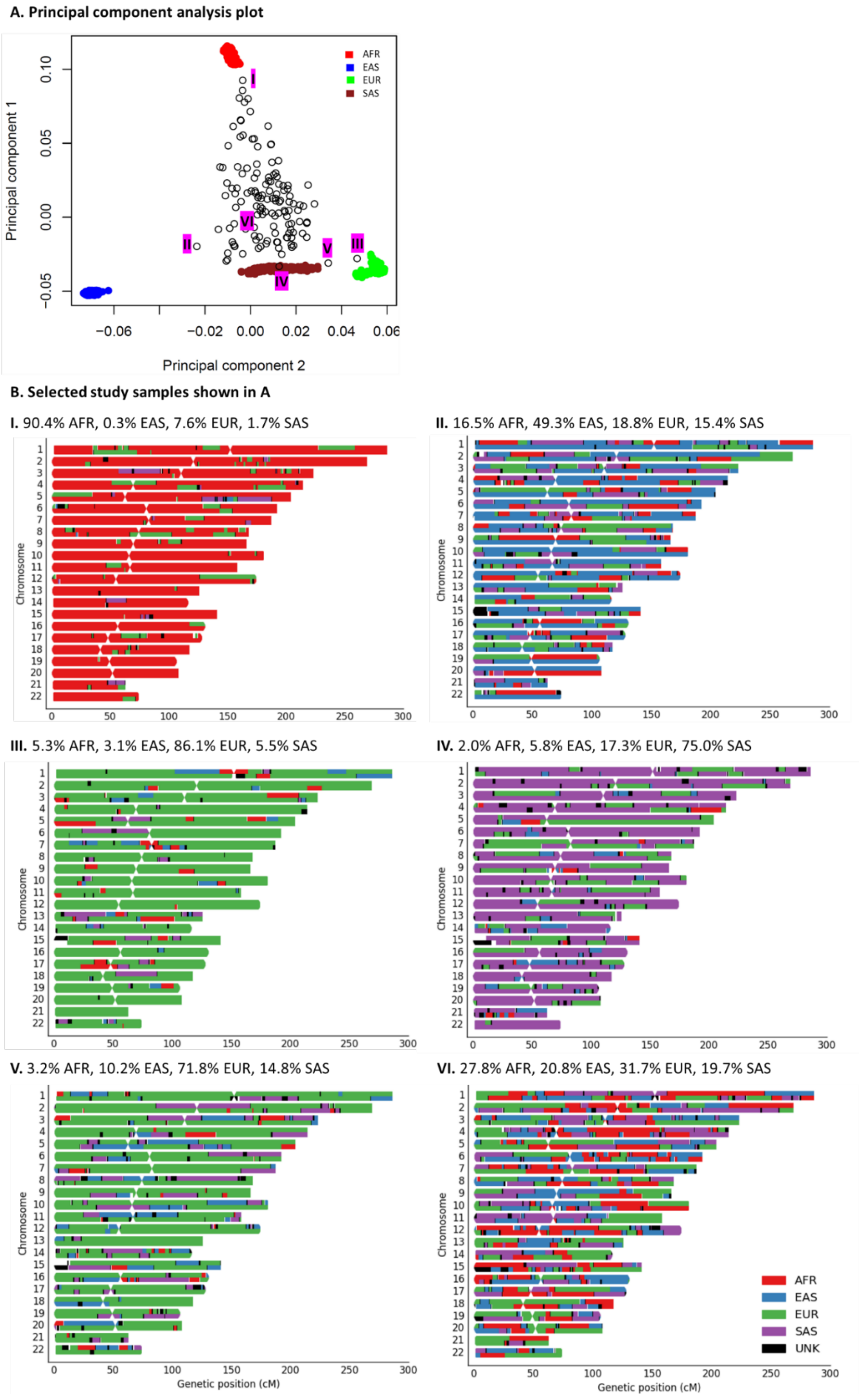

Supplemental Figure 2. Principal component analysis plot and karyograms of selected War-PATH cohort 3 (mixed ancestry South-African) participants. Panel A shows the selected mixed ancestry participants, whose karyograms are shown in **panel B**. These participants were purposely selected for having the most African ancestry (participant I), most East Asian ancestry (participant II), most European ancestry (participant III), most South Asian ancestry (participant IV), mostly European and South Asian ancestries (participant V) and almost an equal proportion of all ancestries (participant VI). AFR = African; EAS = East Asian; EUR = European; SAS = South Asian; and, UNK = unknown, War-PATH = WARfarin anticoagulation in PATients in Sub-Saharan Africa.

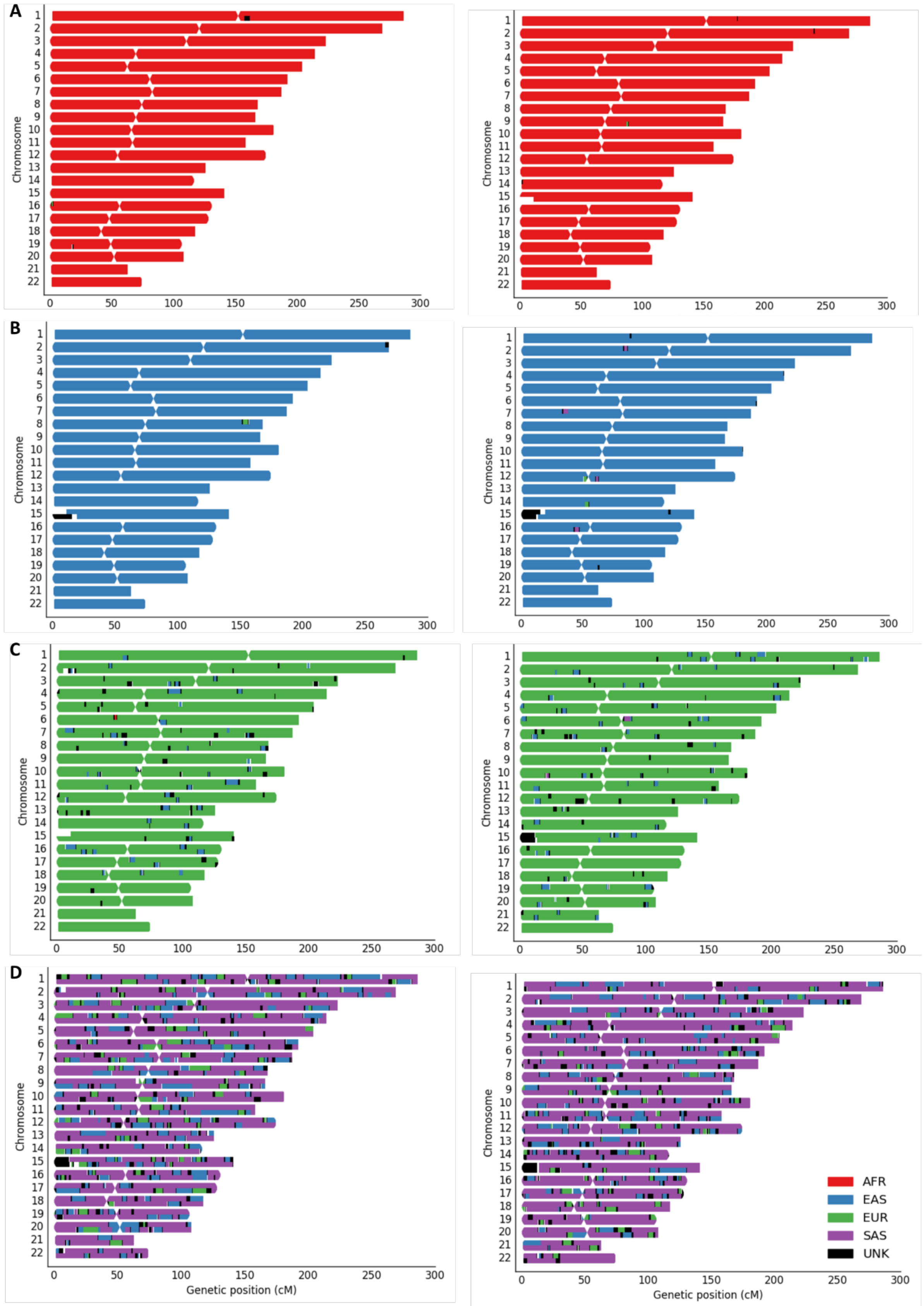

Supplemental Figure 3. Karyograms of selected reference samples. A. African (AFR) samples. **B.** East Asian (EAS) samples. **C.** European (EUR) samples. **D.** South Asian (SAS) samples. Some reference samples, especially those from South Asia were admixed (e.g. containing other ancestries). The --reanalyze-reference flag, which recalculates admixture in the reference samples and improves the ability to distinguish ancestries in the study samples[3 4] was therefore used. UNK = unknown.

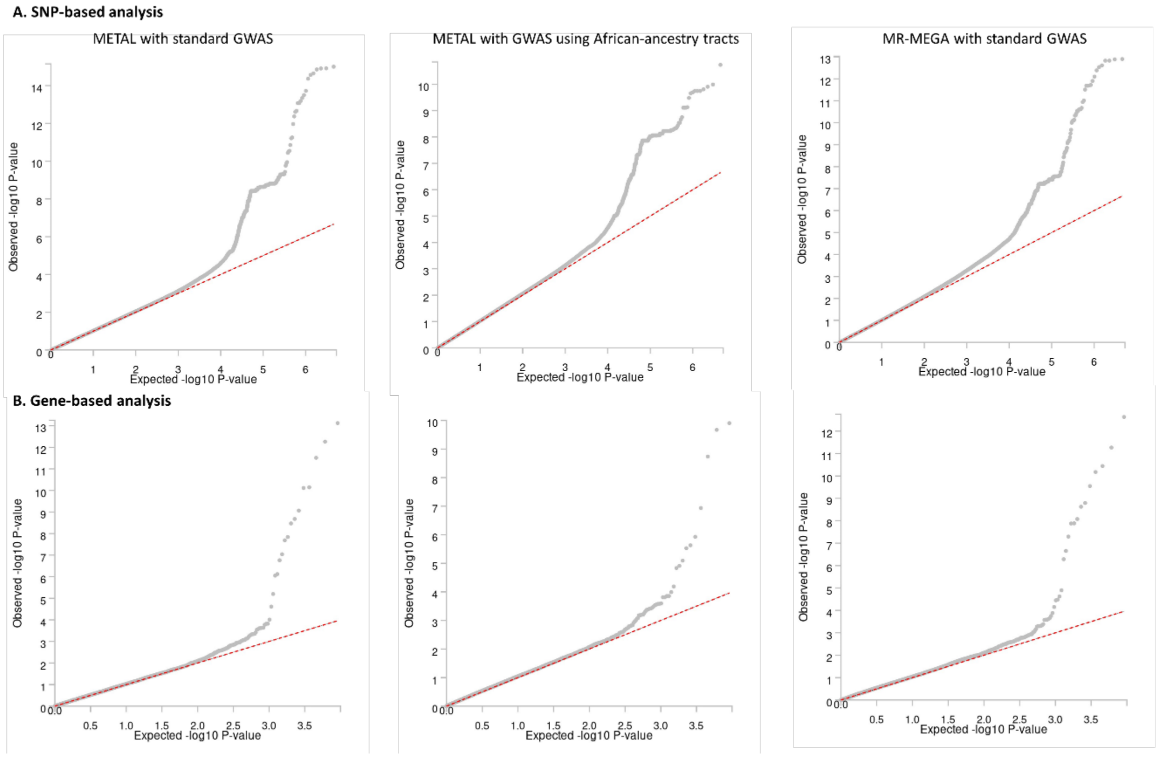

Supplemental Figure 4. Quantile-quantile (QQ) plots of A. SNP-based analysis and B. Gene-based analysis.

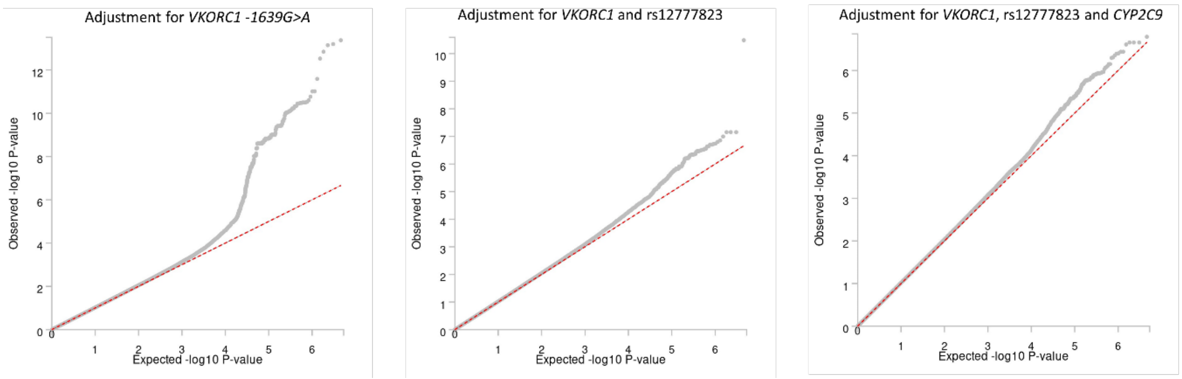

Supplemental Figure 5. Quantile-quantile (QQ) plots the association between SNPs and stable warfarin dose after pooling six African-ancestry cohorts in a meta-analysis (n = 1,504) and conditioning for well-established loci. *CYP2C9* = cytochrome P450, family 2, subfamily C, polypeptide 9; *VKORC1* = vitamin K epoxide reductase complex, subunit 1.

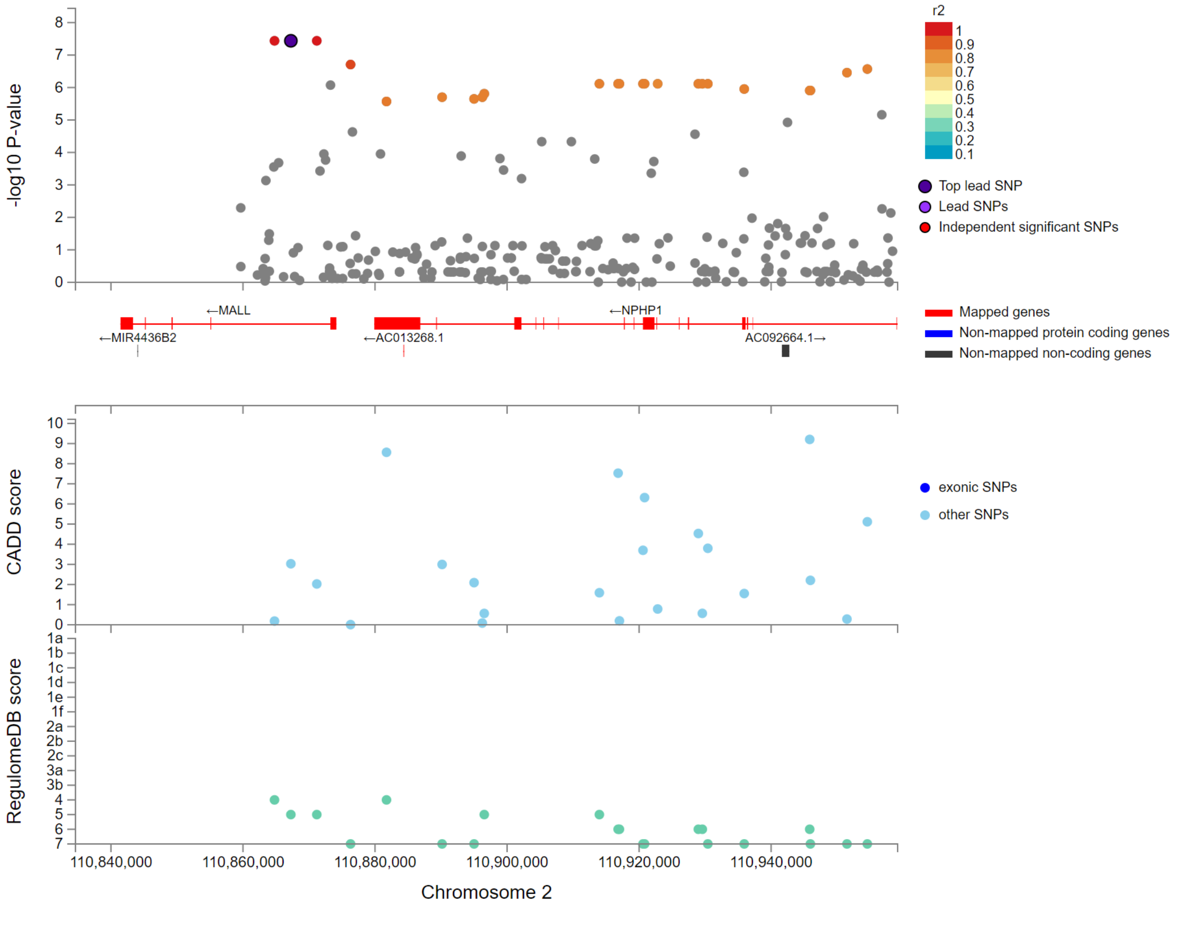

Supplemental Figure 6. Zoom plot for the *MALL* locus. This plot was obtained using the FUMA GWAS platform. eQTL = expression Quantitative Trait Loci, CADD = Combined Annotation Dependent Depletion, FUMA GWAS = Functional Mapping and Annotation of Genome-Wide Association Studies, MALL = mal, T cell differentiation protein like.

Supplemental References

1. Genomes Project C, Auton A, Brooks LD, et al. A global reference for human genetic variation. *Nature* 2015;526(7571):68-74. doi: 10.1038/nature15393 [published Online First: 2015/10/04]
2. Patterson N, Petersen DC, van der Ross RE, et al. Genetic structure of a unique admixed population: implications for medical research. *Hum Mol Genet* 2010;19(3):411-9. doi: 10.1093/hmg/ddp505 [published Online First: 2009/11/07]
3. Atkinson EG, Maihofer AX, Kanai M, et al. Tractor uses local ancestry to enable the inclusion of admixed individuals in GWAS and to boost power. *Nat Genet* 2021;53(2):195-204. doi: 10.1038/s41588-020-00766-y [published Online First: 2021/01/20]
4. Maples BK, Gravel S, Kenny EE, et al. RFMix: a discriminative modeling approach for rapid and robust local-ancestry inference. *Am J Hum Genet* 2013;93(2):278-88. doi: 10.1016/j.ajhg.2013.06.020 [published Online First: 2013/08/06]
